# Correlation of regional surveillance system with changes in epidemiology of HIV infection in Nizhny Novgorod Region, Russia from 2006-2010

**DOI:** 10.1186/1753-6561-7-S1-P1

**Published:** 2013-01-30

**Authors:** Kughan Govinden, OV Kovalishena, NV Saperkin

**Affiliations:** 1 Nizhny Novgorod State Medical Academy, Oblast, Russia

## Background

According to UNAIDS 2011, annual new HIV infections fell by 21% between 1997 and 2010 and improving globally. However, since 2001, HIV prevalence in Russia, Eastern Europe and Central Asia has increased by 250 percent, making the region home to the world’s most rapidly expanding epidemic. It has been widely discussed in Russian National Infectious Disease meetings that the surveillance system of HIV here sometimes is not sufficient.

## Methods

(a) Peer-reviewed articles, conference proceedings and technical reports published from 2006-2010 were reviewed for information regarding inadequate surveillance system in Russian Federation. (b) Information regarding incidence and prevalence rate (per 100000 population), modes of transmission (percentage,%) and distribution by sex (percentage,%) of HIV infection from 2006-2010 for Nizhny Novgorod Region (NNR), Volga Federal Region (VFR) and Russian Federation (RF) is obtained from Centers for Disease Control and Prevention of Nizhny Novgorod Region. (c) Comparative statistical analysis is made using Programs EpiInfo 7 and Microsoft Excel 2007.

## Results

(a) Based on review of articles and journals, HIV surveillance system in the Russian Federation has some disadvantages, for example: sentinel serosurveillance and behavioral surveillance are not conducted regularly,etc. (b) From 2006-2010, incidence rates of HIV infection in NNR increased from 12.4 to 33.8 per 100000 population (p<0.001) and in RF from 31.1 to 46.8 (p<0.001). In VFR, incidence rate during 2006-2009 increased from 31.5-41.5 (p<0.001) but decreased to 34.1 in 2010. (c) Prevalence rates of HIV infection in NNR increased from 125.6 to 225.8 per 100000 population (p<0.001), more drastically in RF from 244.4 to 453.4 (p<0.001) and in Volga FR from 283.6 to 412.1 (p<0.001). (d) Analysis showed that there are increases in placental (*2.3%-3.8%) and sexual pathway (*35.4% to 42.3%) of transmission whereas a decrease in parenteral pathway (*62.3% to 53.9%).*p<0.001 (e) Number of infected males increased from 56.3% to 60.1% (p<0.001) and infected females decreased from 43.7% to 39.9% (p<0.001).

## Conclusions

Insufficient surveillance system can result in high number of incidence rates in NNR, VFR and RF and pronounced increasing trend of prevalence rate in VFR and RF from 2006-2010. The disadvantages in surveillance system can also be related to increases in placental and sexual modes of transmission and infected male sex distribution.

